# Variation of Daily Care Demand in Swiss General Hospitals: Longitudinal Study on Capacity Utilization, Patient Turnover and Clinical Complexity Levels

**DOI:** 10.2196/27163

**Published:** 2021-08-19

**Authors:** Narayan Sharma, René Schwendimann, Olga Endrich, Dietmar Ausserhofer, Michael Simon

**Affiliations:** 1 Institute of Nursing Science Department of Public Health Faculty of Medicine, University of Basel Basel Switzerland; 2 Patient Safety Office University Hospital Basel Basel Switzerland; 3 Directorate of Medicine Inselspital University Hospital Bern Bern Switzerland; 4 College of Health-Care Professions Claudiana Bozen Italy; 5 Nursing Research Unit Inselspital University Hospital Bern Bern Switzerland

**Keywords:** inpatient population, routine data, general hospitals, capacity utilization, clinical complexity, patient data, hospital system, complexity algorithm

## Abstract

**Background:**

Variations in hospitals’ care demand relies not only on the patient volume but also on the disease severity. Understanding both daily severity and patient volume in hospitals could help to identify hospital pressure zones to improve hospital-capacity planning and policy-making.

**Objective:**

This longitudinal study explored daily care demand dynamics in Swiss general hospitals for 3 measures: (1) capacity utilization, (2) patient turnover, and (3) patient clinical complexity level.

**Methods:**

A retrospective population-based analysis was conducted with 1 year of routine data of 1.2 million inpatients from 102 Swiss general hospitals. Capacity utilization was measured as a percentage of the daily maximum number of inpatients. Patient turnover was measured as a percentage of the daily sum of admissions and discharges per hospital. Patient clinical complexity level was measured as the average daily patient disease severity per hospital from the clinical complexity algorithm.

**Results:**

There was a pronounced variability of care demand in Swiss general hospitals. Among hospitals, the average daily capacity utilization ranged from 57.8% (95% CI 57.3-58.4) to 87.7% (95% CI 87.3-88.0), patient turnover ranged from 22.5% (95% CI 22.1-22.8) to 34.5% (95% CI 34.3-34.7), and the mean patient clinical complexity level ranged from 1.26 (95% CI 1.25-1.27) to 2.06 (95% CI 2.05-2.07). Moreover, both within and between hospitals, all 3 measures varied distinctly between days of the year, between days of the week, between weekdays and weekends, and between seasons.

**Conclusions:**

While admissions and discharges drive capacity utilization and patient turnover variation, disease severity of each patient drives patient clinical complexity level. Monitoring—and, if possible, anticipating—daily care demand fluctuations is key to managing hospital pressure zones. This study provides a pathway for identifying patients’ daily exposure to strained hospital systems for a time-varying causal model.

## Introduction

Hospitals are constantly challenged by changing patient care demands. If this outweighs available resources, it can affect the quality of care and patient safety [1]. Demand factors include daily patient volume, turnover, and clinical complexity of patients requiring diagnoses and therapies [1,2]. Responding to variations in any of these factors, hospitals adjust their resource supplies (eg, by changing shift-level staffing or resources for each day, between workdays and weekends, for different seasons, and throughout the year) [3,4].

Capacity utilization, which is based on the number of beds occupied vs those available [5], offers one perspective to view hospital care demand [2,6,7]. Over recent decades, most health care systems’ capacity utilization has increased, while total numbers of available beds have decreased [3,8]. This trend mainly reflects policies to reduce health care costs and to increase efficiency (eg, by the use of diagnostic-related groups [DRGs]) [8,9].

If capacity utilization is too high (eg, above 80% or 85%), it might overburden health care systems and their workforces [10,11], possibly leading to adverse patient outcomes such as infections or even death [5,12,13]. Capacity utilization is high—exceeding 90%—in Canada, Israel, and Ireland, followed by the United Kingdom, Norway, and Switzerland, all of which report figures above 80% [14]. Within a country, capacity utilization also varies between hospital types, geographic regions, and populations served [6,15]. In Switzerland, the most recent annual capacity utilization figures for acute care hospitals were between 70% and 82% [16,17].

As noted above, care demand also relies on patient turnover and patient complexity [18]. Patient turnover refers to the admission and the discharge or transfer of patients between units or hospitals [19], requiring resources [20]. “Census variability,” “churn,” or “environmental turbulence” cover the same or similar concepts [21]. Patient complexity refers to the severity or complexity of each patient’s clinical needs. For instance, patients admitted to the intensive care unit generally require more resources than those in a general ward, representing a resource-intensive caseload.

Disease severity is commonly measured via the Charlson-Elixhauser comorbidity or case-mix index; however, this does not include all relevant clinical conditions or morbidities, and its interpretation is commonly influenced by reimbursement policies or the cost of medication and treatment [22-24]. One alternative measure is the patient clinical complexity level. As part of the German DRG system, the patient clinical complexity level reflects upon not only complications and comorbidities but also their levels of clinical severity on a 5-point scale (ie, 0=no, 1=mild, 2=moderate, 3=severe, and 4=very severe clinical complexity) [25]. A complex algorithm, depending on primary and secondary diagnoses and estimated severities, allows determination of the cumulative effect of the diagnoses per treatment episode [22,26,27]. A higher patient clinical complexity level indicates a more complex and resource-intensive caseload.

As capacity utilization, patient turnover, and patient clinical complexity level all offer necessary perspectives on hospital care demand, all 3 are vital for optimal resource allocation [28]. All 3 are also connected; for instance, complex patients usually stay longer in hospitals, increasing capacity utilization. Furthermore, with each transfer or referral to another hospital, additional load is created, as patients need to be assessed at admission or be prepared for discharge [21]. Understanding these factors’ daily variation is a vital step toward optimizing health care structures and processes [29]. Regarding the daily variability of care demand, analysis of long-term data can help to anticipate when staffs or supplies will be depleted or strained, thereby, indicating when and where to allocate resources. Particularly, the traditional measures of capacity utilization (eg, midnight count [29,30]) and patient turnover (eg, the inverse of the length of hospital stay [31,32]) may not convey the dynamic nature of actual daytime hospital care demands [33]; certainly, neither incorporates patient complexity and severity.

Additionally, the valid, highly granular longitudinal (daily or weekly) measurement of care demand offers a precise and in-depth view of how care needs fluctuate and evolve. As capacity utilization, patient turnover, and patient clinical complexity level are time-sensitive variables, the 3 together offer great potential to accurately represent daily care demand dynamics. Such information should enable health care managers to anticipate capacity needs to accommodate patients during a typical weekday, weekend, or seasonal peak [3].

Therefore, this study aimed to describe the daily care demand in general hospitals from a longitudinal perspective, specifically, the daily peaks and variations during weeks (ie, weekends vs weekdays), as well as seasons. This study describes the daily variability of (1) capacity utilization, (2) patient turnover, and (3) patient clinical complexity levels of Swiss general hospitals’ inpatient populations.

## Methods

### Study Design, Setting, and Population

**Figure 1:**
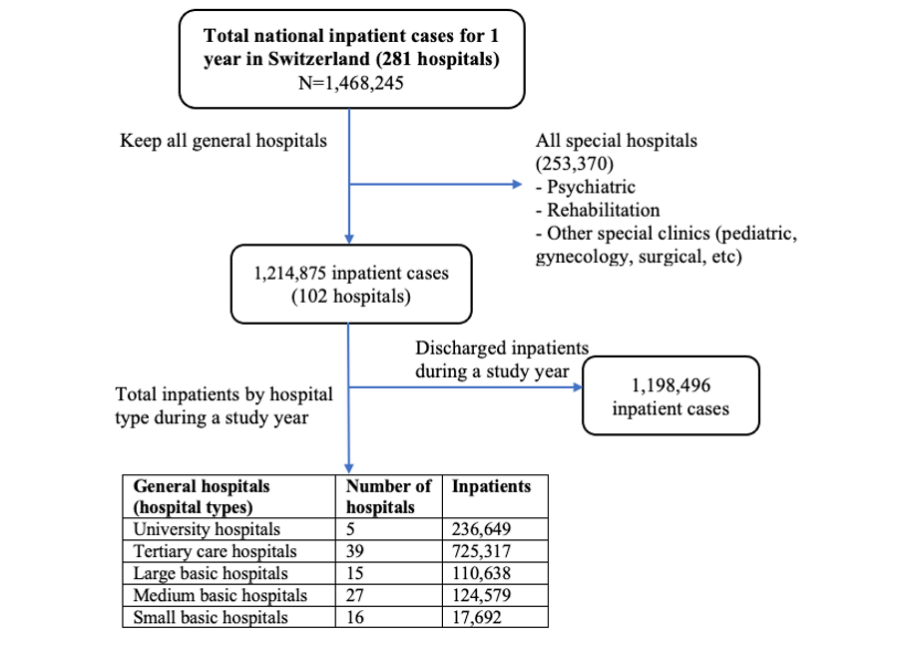
Flow diagram of inpatient cases for the analysis.

This is a retrospective, population-based analysis using 1 year of hospital data extracted from the 6-year (2012-2017) dataset obtained from the Swiss Federal Statistics Office (FSO). Based on a data protection contract (as stipulated by article 22 of the Swiss Federal Act on Data Protection), the FSO provided anonymized data from all Swiss hospital inpatients hospitalized over the study period. The data covered general as well as specialized care facilities such as pediatric, gynecological, psychiatric, and rehabilitative hospitals [34]. The FSO divides general hospitals into 5 classifications: university hospitals, tertiary care hospitals, large basic hospitals, medium basic hospitals, and small basic hospitals. Classification is based on the number of cases treated per year and a weighted sum of service points (based on a combination of the number of hospital units and the levels of care delivered) assigned by the Swiss Medical Association [17,34]. For instance, based on the Swiss Medical Association classification, a university hospital requires the weighted sum of service points to be >100 units and >30,000 cases per year [34]. For this study, we included a 1-year patient population dataset from general hospitals to limit interhospital heterogeneity. Due to the Swiss Data Protection Act’s stipulations regarding patient anonymity, we were unable to trace patients across years. An overview of the process of selecting inpatient cases for analysis is included in the flow diagram in Figure 1.

### Dataset and Study Variables

We extracted 1-year datasets, including (1) all inpatients discharged during each study year, regardless of admission date, and (2) all inpatients admitted during each study year, regardless of discharge date. From the full FSO hospital dataset, we also extracted data for all relevant routine administrative and clinical variables for the study period (Multimedia Appendix 1).

### Statistical Analyses

All statistical analyses were conducted using R, version 3.6.3 for Mac OS [35]. The following statistical packages were used: (1) dplyr [36] and tidyr [37] for data preparation; (2) lubridate [38] and stringr [39] for handling time and date; and (3) ggplot2 [40], patchwork [41], and scales [42] for plotting.

#### Descriptive Overview

For each hospital type, in addition to the number of hospitals, the total numbers of patients, admissions, and discharges were recorded by gender in frequencies and percentages. Length of stay (LOS), in days, was calculated for all patients by subtracting each individual’s admission date from their discharge date. The results were aggregated at the level of the hospital type and presented as mean (95% CI) and median (IQR), for each type.

#### Capacity Utilization Measure

For each hospital, each study day’s capacity utilization was calculated as a percentage, using that hospital’s highest recorded daily bed occupancy for that year as the denominator [5,9]. Each patient’s admission and discharge dates were used to calculate the number of patients present each day in each hospital. The capacity utilization of a day in a given hospital included all patients admitted or discharged on that day and the number who were present the previous day and not discharged or deceased. As noted above, patients admitted before the study year (eg, in December of the previous year) were included in the study until their discharge. Patients not discharged during the study year were included until the end of the study year. Total capacity utilization was calculated for each hospital (n=102) for 365 days (with a total of 37,230 time points).

Furthermore, daily percentages of capacity utilization were summarized by hospital type, along with means (95% CIs), SDs, and minimum-maximum values. To visualize daily variations in capacity utilization, smoothed lines were plotted, with the 95% CIs around the mean, for each hospital type. For each hospital type, we also plotted graphs to show variation by day of the week. Finally, for each hospital type, weekday vs weekend variations were plotted for each week of the year. There was no dot plotted for weekdays of the first week, as the first day of this study year was a weekend (Sunday).

#### Patient Turnover Measure

As calculated for capacity utilization, daily patient turnover was calculated for each hospital and aggregated by hospital type. The patient turnover rate was calculated as absolute counts of admissions, discharges, and deceased patients for a particular day divided by the total number of patients for that day per hospital [21]. As opposed to using the inverse of the average LOS method, this approach has the advantage of adequately representing the volume of activity either for entire days or short hospital stays as contributors to increasing patient throughput [19,21,43]. The percentage of patient turnover per day was calculated and further summarized by hospital type as mean (95% CI), SD, and minimum-maximum figures. To display daily variations in patient turnover, similar displays were plotted for them as for capacity utilization.

#### Patient Clinical Complexity Level

Our patient clinical complexity level data covered only patients discharged during the study year (as the International Classification of Diseases [ICD]-10 codes were not available for patients until they were discharged), and Swiss DRG version 6 was applied for that study year [44]. The patient clinical complexity level calculation was based on a complex algorithm, providing clinical complexity and comorbidity level values (ranging from 0-4) for all possible primary and secondary diagnoses per patient case [26]. Developed as part of the complexity and comorbidity level Refinement Project in Australia [26,27,45], this algorithm was applied to determine each patient’s final patient clinical complexity level. To facilitate this process, we used the grouping system provided by Swiss DRG AG [44].

We began by organizing our data input into a readable format via the grouping system. We chose the “SwissDRG Batchgrouper Format 2017” short input format, which provides an anonymous case identifier, plus the patient’s age, sex, admission and discharge date, LOS, primary and secondary diagnoses, and treatment procedure codes. “DRG Output format for SwissDRG” results were then obtained, including patient clinical complexity level values for each case. Individual patient clinical complexity level values were further transformed to average daily patient clinical complexity level values per hospital, using each patient’s admission and discharge dates (ie, each case’s patient clinical complexity level value is applied to each day for that hospital until discharge). Daily patient clinical complexity level values were further summarized by hospital type as means (95% CIs), SDs, and minimum-maximum figures. As for the other 2 measures, similar displays were plotted to display variation in daily patient clinical complexity levels.

## Results

### Descriptive Overview

During the study year, 1,214,875 inpatients stayed in the 102 Swiss general hospitals, of which 16,379 cases (1.35%) continued to the following year. Of the 1,214,875 inpatients, the 5 university hospitals covered 19.50% (n=236,649), the 39 tertiary care hospitals covered 59.70% (n=725,317), and the 58 basic hospitals covered 20.81% (n=252,909) of the patient population. Overall, of the 1,214,875 inpatients, there were approximately 6.87% (n=91,078) more female than male patients. The average patient LOS across all general hospitals was 6.43 (95% CI 6.40-6.46) days; the median LOS was 3.7 (IQR 2.0-7.0) days. The general characteristics of the study population by hospital type are presented in Multimedia Appendix 1.

### Variation of Daily Capacity Utilization

Average daily capacity utilization ranged from 527-2340 patients in university hospitals, 87-1099 patients in tertiary care hospitals, 16-304 patients in large basic hospitals, 7-179 patients in medium basic hospitals, and 1-93 patients in small basic hospitals. Notably, 3 small basic hospitals had average daily capacity utilization numbers below 10 patients.

Across the study period, the average daily capacity utilization was highest in university hospitals and the lowest in small basic hospitals (Table 1). However, the range (ie, the variation between the lowest and the highest daily capacity utilizations for a study year) was almost 98% (eg, 1.7-100.0) for small basic hospitals, 92% for medium basic hospitals, 87% for large basic hospitals, 73% for tertiary care hospitals, and 44% for university hospitals.

**Table 1:** Daily capacity utilization, patient turnover, and patient clinical complexity level per hospital, by hospital type, from the 1-year patient population.

Hospital type (median^a^)	Capacity utilization (%)			Patient turnover^b^ (%)				Patient clinical complexity level (0-4^c^)		
	Mean (SD)	95% CI	Min-max^d^	Mean (SD)	95% CI	Min-max	Mean (SD)		95% CI	Min-max
University hospitals (988)	87.7 (7.7)	87.3-88.0	55.8-100.0	22.5 (7.6)	22.1-22.8	5.7-38.7	2.06 (0.2)		2.05-2.07	0.81-2.57
Tertiary care hospitals (298)	78.7 (10.2)	78.5-78.9	27.3-100.0	28.8 (7.5)	28.6-28.9	2.7-54.6	1.78 (0.3)		1.78-1.79	0.42-2.75
Large basic hospitals (120)	71.3 (13.4)	70.9-71.6	13.1-100.0	32.6 (9.2)	32.4-32.9	0.0-75.4	1.46 (0.4)		1.45-1.47	0.09-2.50
Medium basic hospitals (71)	65.3 (15.2)	65.0-65.6	5.9-100.0	34.5 (10.7)	34.3-34.7	0.0-109.1	1.26 (0.6)		1.25-1.27	0.00-2.93
Small basic hospitals (19)	57.8 (22.2)	57.3-58.4	1.7-100.0	24.3 (22.0)	23.8-24.9	0.0-200.0	1.65 (0.8)		1.63-1.67	0.00-4.00

^a^Median number of beds used per day per hospital, by hospital type.

^b^Patient turnover is the percentage of total patients admitted and discharged in a day.

^c^0=No clinical complexity, 1=mild clinical complexity, 2=severe clinical complexity, and 4=very severe clinical complexity.

^d^Min-max: Minimum-maximum value on hospital level within hospital type.

**Figure 2:**
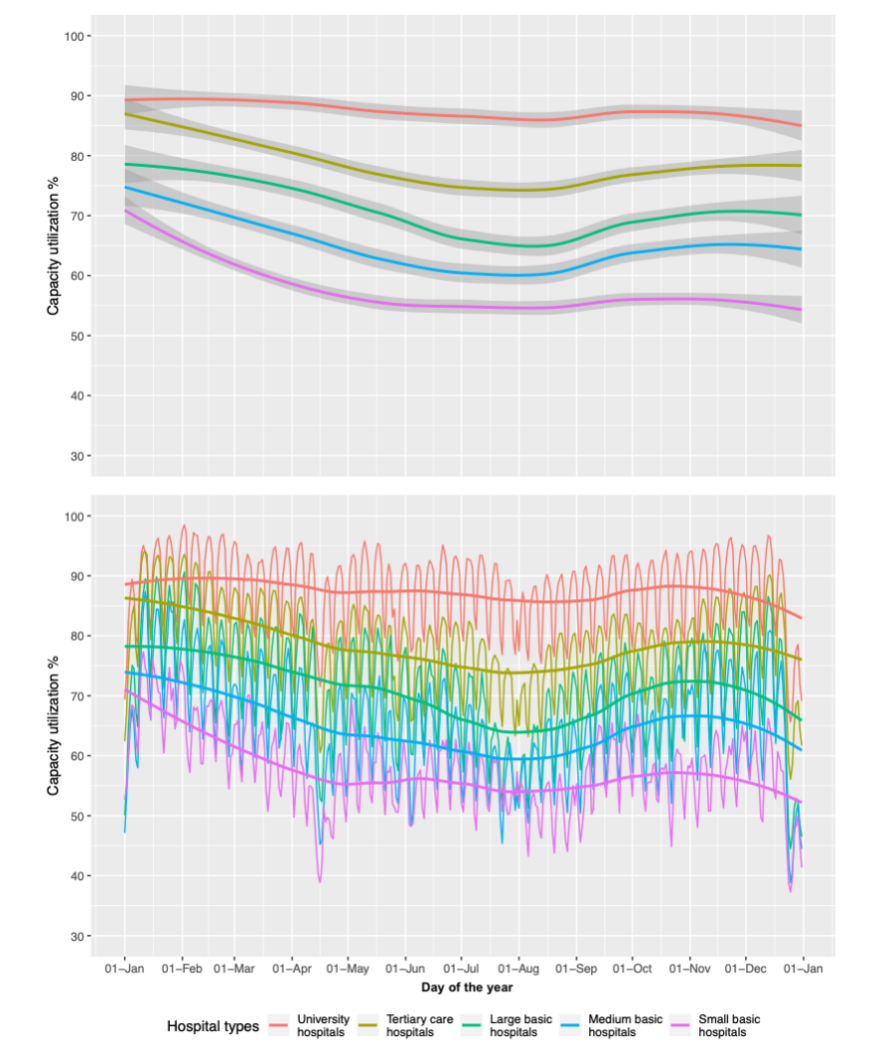
Daily capacity utilization of Swiss general hospital types for 1 year (smooth curve with mean between CIs and line chart).

As indicated by the smooth curves and line charts by hospital categories, university hospitals’ daily capacity utilizations were high throughout the year. Among all hospital types, capacity utilization was lower throughout the summer months (June-August; Figure 2).

**Figure 3:**
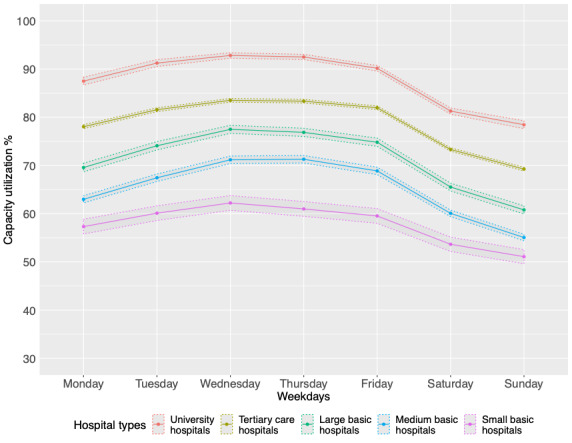
Capacity utilization of Swiss general hospital types with mean between CI by day of the week.

There was a gradual increase in capacity utilization through the early days of the week (Mondays-Wednesdays; Figure 3), followed by a gradual decrease from Fridays to Sundays, across all hospital types. There was roughly a 10% difference in capacity utilization during weekdays than on the weekend, in all hospital types (Table 2).

**Table 2:** Daily capacity utilization, patient turnover, and patient clinical complexity level by weekday vs weekend from the 1-year patient population.

Day by hospital type		Capacity utilization (%)					Patient turnover (%)				Patient clinical complexity level (0-4^a^)			
		Mean (SD)	95% CI			Min-max^b^	Mean (SD)	95% CI	Min-max	Mean (SD)		95% CI	Min-max	
**University hospitals**														
	Weekday	90.8 (3.8)	89.8-91.9			72.8-96.2	25.5 (1.4)	25.1-25.9	16.6-26.6	2.04 (0.21)		2.03-2.05	1.22-2.57	
	Weekend	79.8 (3.0)	78.9-80.6			69.1-85.0	14.8 (0.8)	14.6-15.1	10.5-17.6	2.1 (0.27)		2.08-2.13	0.81-2.55	
**Tertiary care hospitals**														
	Weekday	81.7 (5.6)	80.1-83.3			63.5-92.1	31.9 (1.6)	31.4-32.3	22.2-33.2	1.76 (0.28)		1.75-1.77	0.84-2.69	
	Weekend	71.2 (4.5)	69.9-72.4			61.6-80.9	21.1 (1.1)	20.8-21.4	14.4-24.3	1.83 (0.30)		1.82-1.84	0.42-2.75	
**Large basic hospitals**														
	Weekday	74.6 (7.4)	72.5-76.6			49.6-86.8	35.9 (1.9)	35.4-36.4	25.2-37.9	1.44 (0.38)		1.42-1.45	0.14-2.25	
	Weekend	63.0 (5.9)	61.4-64.6			46.5-74.4	24.4 (1.6)	23.9-24.8	16.7-29.5	1.52 (0.38)		1.5-1.54	0.09-2.5	
**Medium basic hospitals**														
	Weekday	68.4 (7.0)	66.4-70.3			45.8-82.0	37.1 (1.3)	36.8-37.5	31.2-39.3	1.24 (0.54)		1.22-1.25	0.05-2.79	
	Weekend	57.6 (5.5)	56.1-59.1			44.5-70.0	27.8 (1.7)	27.4-28.3	24.0-34.6	1.31 (0.57)		1.29-1.33	0.00-2.93	
**Small basic hospitals**														
	Weekday	60.0 (5.9)	58.3-61.6			44.6-74.1	27.0 (2.4)	26.3-27.6	22.2-31.7	1.63 (0.80)		1.6-1.65	0.00-4.00	
	Weekend	52.2 (5.3)	50.8-53.7			41.4-65.7	18.0 (4.0)	16.9-19.1	11.3-28.8	1.69 (0.81)		1.65-1.73	0.00-4.00	

^a^0=No clinical complexity, 1=mild clinical complexity, 2=severe clinical complexity, and 4=very severe clinical complexity.

^b^Min-max: Minimum-maximum.

**Figure 4:**
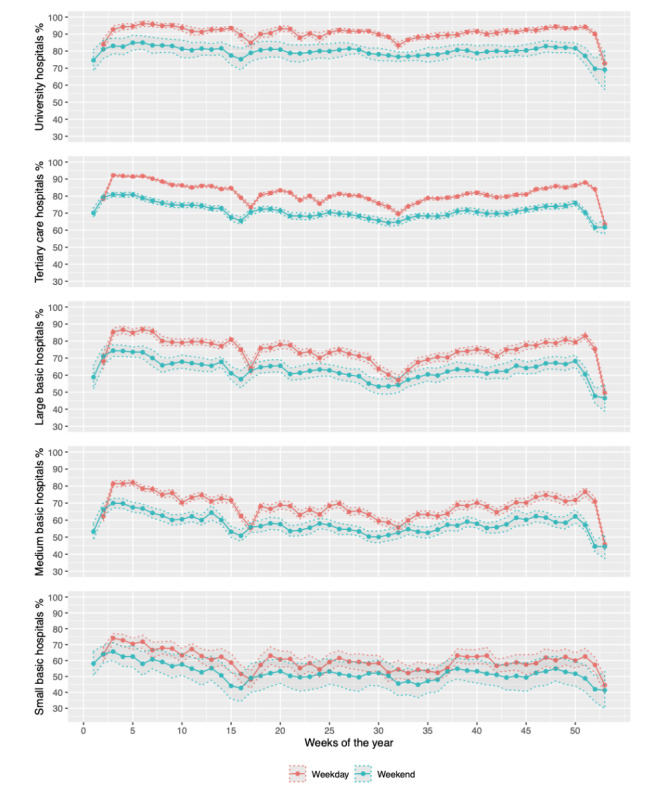
Capacity utilization of Swiss general hospital types with mean between CI by weekday vs weekend for 1 year.

Comparing weekdays with weekends, variations in capacity utilization for each week are shown in Figure 4. With very few exceptions (eg, the 2nd, the 17th, and the final week of the year), the weekly capacity utilization for weekdays was higher than for weekends. These weekly graphs also show lower capacity utilization during the summer months (weeks 20-35).

### Variation of Daily Patient Turnover

Throughout the year, daily patient turnover ranged from 72-468 patients for university hospitals, 5-318 patients for tertiary care hospitals, 0-91 patients for large basic hospitals, 0-78 patients for medium basic hospitals, and 0-33 patients for small basic hospitals. The minimum value of 0 indicates that some hospitals saw neither admissions nor discharges on some days.

During the study year, the mean daily patient turnover percentage was highest in medium-sized basic hospitals and lowest in university hospitals (Table 1). The difference in daily patient turnover range was highest for small basic hospitals, decreasing with each increase in hospital size class. And, as illustrated in the smooth and line chart, daily turnover varied the least in university hospitals and the most in medium basic hospitals (Multimedia Appendix 2).

Exploring the frequency of change in patient movement by day of the week (Multimedia Appendix 2), we found that turnover was highest on Mondays and lowest during weekends. Differences in patient turnover during weekdays and weekends across the 5 hospital types are shown in Table 2. Across the 5 hospital types, daily patient turnover was almost 10% higher on weekdays than on weekends.

The variation of patient turnover for each day of the week and for weekdays vs weekends is shown in Multimedia Appendix 2. In small basic hospitals, with few exceptions (eg, the 31st and 52nd weeks), the mean daily patient turnover was higher for weekdays than for weekends, for all hospital types. Overall patient turnover was lower during the holiday seasons (ie, weeks 17-18 and the final 2 weeks of the year).

### Variation of Daily Patient Clinical Complexity Level

Overall, of the total discharged patients (1,198,496), 10.97% (n=131,442) of patients had severe clinical complexity (patient clinical complexity level 4), while roughly 60.97% (n=730,893) had no clinical complexity (patient clinical complexity level 0; Table 3).

**Table 3:** Patient clinical complexity level by hospital type (N=1,198,496).

Hospital type (N)	Patient clinical complexity level (0-4), n (%)				
	0: No clinical complexity, 730,893, (60.98)	1: Mild clinical complexity, 17,579 (1.47)	2: Moderate clinical complexity, 136,453 (11.39)	3: Severe clinical complexity, 182,129 (15.20)	4: Very severe clinical complexity, 131,442 (10.97)
University hospitals (n=232,127)	122,567 (52.8)	3281 (1.4)	28,812 (12.4)	41,159 (17.7)	36,308 (15.6)
Tertiary care hospitals (n=715,809)	433,514 (60.6)	11,003 (1.5)	83,374 (11.6)	110,756 (15.5)	77,162 (10.8)
Large basic hospitals (n=109,572)	73,800 (67.4)	1397 (1.3)	10,801 (9.9)	14,590 (13.3)	8984 (8.2)
Medium basic hospitals (n=123,530)	90,284 (73.1)	1679 (1.4)	11,237 (9.1)	12,865 (10.4)	7465 (6.0)
Small basic hospitals (n=17,458)	10,728 (61.5)	219 (1.3)	2229 (12.8)	2759 (15.8)	1523 (8.7)

Throughout the year, mean daily patient clinical complexity level varied across the 5 hospital types. It was highest in university hospitals (2.06, 95% CI 2.05-2.07) and lowest in medium basic hospitals (1.26, 95% CI 1.25-1.27; Table 1). This is depicted in the smooth and line chart for the 5 general hospital types (Multimedia Appendix 3). Mean patient clinical complexity level gradually decreased from Monday until midweek but remained highest during the weekend—the opposite of the usual pattern of capacity utilization and patient turnover explored by the days of the week (Multimedia Appendix 3).

Weekday and weekend differences in patient clinical complexity level for the 5 general hospital types are shown in Table 2. Across all hospital types, patient clinical complexity level was almost 0.07 points higher during the weekend than on weekdays. During weekdays, university hospitals’ average daily patient clinical complexity level was 2.04 (95% CI 2.03-2.05); during the weekend, it was 2.1 (95% CI 2.08-2.13). Weekday vs weekend patient clinical complexity level variation over 1 year is shown in Multimedia Appendix 3. Except for a small number of weeks (eg, the 7th and the 31st weeks in small basic hospitals), across all hospital types, the patient clinical complexity level for weekends was higher than for weekdays. Moreover, except for small basic hospitals, patient clinical complexity level dropped in December. This was partly because patient clinical complexity levels could only be calculated for patients discharged during the study year (ie, ICD diagnostic codes were unavailable for patients not discharged during the year, and anonymity considerations made it impossible to track patients across years). However, mean values both for patient clinical complexity level and for LOS were also lower for patients discharged in November and December, with higher patient clinical complexity level values assigned to patients who remained in the hospital across the years’ end (Multimedia Appendix 4).

## Discussion

### Principal Findings

We examined 1 year of routine patient data from all 102 general hospitals in Switzerland. Average daily capacity utilization varied widely, from 57.8% in small basic hospitals to 87.7% in university hospitals. However, patient turnover was highest, at 34.5%, in medium basic hospitals and lowest, at 22.5%, in university hospitals. Moreover, average daily patient clinical complexity level was highest in university hospitals, at 2.06, and lowest in medium basic hospitals, at 1.26. Surprisingly, in small basic hospitals, patient turnover was lower than in tertiary hospitals or either of the 2 other basic hospital types, both of which also had higher mean patient clinical complexity levels throughout the year. Another interesting finding was that the average daily patient clinical complexity level was highest on weekends. Additionally, all hospital types showed distinct weekday, weekend, and seasonal effects regarding capacity utilization, patient turnover, and patient clinical complexity level.

Concerns have been raised that capacity utilization alone does not explain hospitals’ total care demand [6,18]. Therefore, we viewed this alongside daily volumes of admitted and discharged patients and complexity [18]. This study explored all 3 measures, showing that capacity utilizations and patient clinical complexity levels were highest but patient turnovers were lowest in university hospitals. Even with a large proportion of inpatients in tertiary care institutions, university hospitals generally operate at close to full capacity and with the most complex patient cases. Thus, more care resources need to be allocated to university hospitals [3]. On the other hand, in small basic hospitals, where capacity utilization and patient turnover were relatively low, patient clinical complexity level was above those of the other basic hospitals. This indicates that complex cases are still treated in small basic hospitals, possibly, due to geographic proximity, which may also relate to older patients’ preference for them: across all hospital types, these hospitals have the highest mean patient age. In light of these small basic hospitals’ continued relevance (as they still treat complex cases), they may warrant greater resource allocation.

We measured daily demand for Swiss general hospital care longitudinally for 1 year. As it has also been observed in other studies regarding days of the week, Saturdays and Sundays had the lowest capacity utilization and patient turnover [3,46]. Moreover, we observed that patient clinical complexity level was highest during weekends, possibly, because more complex patients remain in the hospital through the weekend. Comparing weekly demand throughout the year, a clear distinction between weekends and weekdays was shown, with the highest variability occurring in small basic hospitals, possibly indicating suboptimal patient flow. Concurrently, seasonal variations were also seen. Capacity utilization was mainly highest in the winter and relatively low in the summer months, whereas patient turnover was constant throughout the year, dropping off toward the end of the year. However, patient clinical complexity level remained quite constant, with a slight drop during the summer months and a marked reduction during December. These changes tended to correspond with holidays, possibly, including higher patient discharge rates and fewer admissions before the holidays and at the end of the year. The capture of daily patient complexity during the end of the year was also reduced because ICD-10 codes were unavailable for patients who were admitted but not discharged during the study year. Furthermore, patients with higher patient clinical complexity levels were more likely to have longer LOSs, particularly, across the Christmas and end-of-year period.

### Potential Implications

Based on capacity utilization, patient turnover, and patient clinical complexity level, the variability of daily care demand in general hospitals directly impacts resource use. From the perspective of a single hospital, the extent of that impact depends on the degree of variation in care need, as well as on the hospital administrators’ ability to adapt or otherwise respond to changes either in resource supply or demand. Our analysis on the demand dynamics of the Swiss health care system indicates that monitoring of care demand is useful to create surge capacity during disasters or the COVID-19 pandemic [20,47], by offering alternative solutions such as smoothing workloads and coordinating early discharges. It also has the potential to help health system planners and hospital managers to tailor their staffing and other resources to match care demand and the early planning of admissions (eg, surgeries or follow-up treatment) [48], to control patient flow for smoother service use.

What this analysis cannot describe is the human resources and other resources needed to meet care demands (ie, balancing care demands or any other supply-demand chain). To do so would require a full exploration of the relevant human resources (eg, physician and nurse staffing), in the light of each hospital’s care demand. Furthermore, application of time-driven activity-based costing methods could provide a framework to identify process improvements for health care delivery [49,50]. However, we were not able to consider time-driven activity-based costing, as we do not have sufficient data regarding resource consumption (eg, personnel, equipment, and supplies) during the patients’ journey along the clinical pathway [49].

Some studies have linked higher capacity utilization and patient turnover with adverse patient outcomes [5,21]. In addition to these results, acknowledging the effect of clinical complexity alongside capacity utilization and patient turnover might bring us closer to understanding the factors that stress hospital systems and the effects that a stressed system have on patient outcomes such as in-hospital mortality. Describing daily care demand to identify meaningful variation will require further studies (eg, examining patients’ time-varying exposure to hospitals or units under pressure and the impact on the quality-of-care indicators and patient outcomes in causal models). Particularly, extending previous research on capacity utilization and in-hospital mortality [5,51] and using daily capacity utilization as time-varying exposure (ie, systemic stress factor), it would be of interest to explore the daily patient turnover and patient clinical complexity level as time-varying confounders. In a practical sense, this might also allow monitoring of pressure zones (eg, to manage care demand, where possible) in hospitals, which could reduce avoidable adverse events or death [5].

### Strengths and Limitations

To our knowledge, this was the first study to explore hospital care demand dynamics via daily measurements of capacity utilization, patient turnover, and patient clinical complexity level on a national health system level. Furthermore, applying the standard methodology, programming, and software for large datasets allowed a longitudinal perspective by computing and visualizing demand dynamics per day of the year, day of the week, and weekdays vs weekends.

This study also had notable limitations. While we explored demand dynamics in detail, we could not do so with supply dynamics (eg, staffing, resources, etc)—an entire category of critical information in the demand-supply equation. Due to the large sample and FSO data composition (ie, aggregated data), it was also not possible to explore demand dynamics at the unit level—the interface between patients’ care demand and health professionals’ provision of care. Also, as we used codes assigned in routine data, the patient diagnoses and other variables could be biased by factors such as the accuracy of physicians’ and nurses’ documentation, lack of availability of ICD-10 codes for patients who were not discharged, and intentional upcoding of diagnoses to more expensive Swiss DRG categories [52,53].

### Conclusions

This study illustrates daily care demand based on capacity utilization, patient turnover, patient clinical complexity level, and the variability of these factors between the 5 classes of Swiss general hospitals. For all 5 types, our analyses indicated distinct differences in capacity utilization, patient turnover, and patient clinical complexity level between days of the week, weekdays vs weekends, and seasons. This longitudinal study is a step toward detecting possible variables to be considered for time-varying exposure (eg, capacity utilization) and confounders (eg, patient clinical complexity level) in developing a casual model of tipping points and their links with quality of care or patient outcomes. Essentially, the variability of care demand provides a new perspective for gauging when hospitals are under strain, and this could help avoid pressure zones with a combination of appropriate resource allocation and care-demand planning in general hospitals.

## Appendix

Multimedia Appendix 1 Description of variables and general characteristics of the study population for 5 Swiss general hospital types.

Multimedia Appendix 2 Percentage of patient turnover by days of the year, days of the week (Monday to Sunday), and weekdays vs weekends over 1 year for 5 Swiss general hospital types.

Multimedia Appendix 3 Average patient clinical complexity levels by days of the year, days of the week (Monday to Sunday), and weekdays vs weekends over 1 year for 5 Swiss general hospital types.

Multimedia Appendix 4 Distribution of patient clinical complexity levels for the last 10 weeks of the year with the length of stay and average inpatients on weekly basis (showing complex patient stays over Christmas and the end of the year).

## Abbreviations

DRG: diagnostic-related group
FSO: Federal Statistics Office
ICD: International Classification of Diseases
LOS: length of stay
