# Supplementary material for: Variation of Daily Care Demand in Swiss General Hospitals: Longitudinal Study on Capacity Utilization, Patient Turnover and Clinical Complexity Levels
Source: J Med Internet Res. 2021 Aug 19;23(8):e27163. doi: 10.2196/27163 (PMC8414292; doi:10.2196/27163)
Supplement: Multimedia Appendix 1 [file jmir_v23i8e27163_app1.pdf]

Multimedia Appendix 1

Description of variables and general characteristics of the study population for five Swiss general hospital types.

Table A. Description of the study variables

Variables	Short description
Case identifier	Unique anonymous numeric identifier for each case
Age	Age of patient in five-year groups
Sex	Patient’s sex
Hospital identifier	Unique anonymous numeric identifier for each hospital
Hospital type	Hospital FSO classification code
Admission date	Patient’s date of hospital admission
Discharge date	Patient’s date of discharge from hospital
Primary Diagnosis	ICD-10 GM primary patient diagnosis code
Secondary diagnosis	ICD-10 GM secondary patient diagnosis code
Procedure	CHOP code for procedure taken for patient

FSO: Federal Statistics Office; ICD-10 GM: International Classification of Diseases- version 10 German Modification; CHOP: Swiss Operation Classification

Table B. General characteristics of the study population

Hospital Types	Number of hospitals N (%)	Inpatients (%) N = 1,214,875 Males	Females	Discharges (%) N = 1,198,496 Males	Females	Length of stay (days) N = 1,198,496 Mean (95% CI)	Median (IQR)
University Hospitals	5 (4.9)	116,143 (20.5)	120,506 (18.6)	113,910 (20.4)	118,217 (18.5)	8.76 (8.65–8.86)	4.12 (2.04–9.12)
Tertiary care hospitals	39 (38.2)	339,551 (60.0)	385,766 (59.4)	335,185 (60.1)	380,624 (59.4)	6.06 (6.04–6.08)	3.67 (2.04–6.96)
Large basic hospitals	15 (14.7)	49,930 (8.8)	60,708 (9.4)	49,449 (8.9)	60,123(9.4)	5.33 (5.29–5.37)	3.33 (1.96–5.96)
Medium basic hospitals	27 (26.5)	52,955 (9.4)	71,624 (11.0)	52,501(9.4)	7,1029 (11.1)	4.93 (4.9–4.97)	3.25 (1.96–5.38)
Small basic hospitals	16 (15.7)	7,122 (1.3)	10,570 (1.6)	7,051 (1.3)	10,407 (1.6)	7.67 (7.52–7.82)	4.12 (2.04–9.12)
Total	102 (100)	565,701 (100)	649,174 (100)	558,096 (100)	640,400 (100)	6.43 (6.40–6.46)	3.7 (2.00–7.00)

CI: Confidence Interval, IQR: Intra-Quartile Range
