# Supplementary material for: Variation of Daily Care Demand in Swiss General Hospitals: Longitudinal Study on Capacity Utilization, Patient Turnover and Clinical Complexity Levels
Source: J Med Internet Res. 2021 Aug 19;23(8):e27163. doi: 10.2196/27163 (PMC8414292; doi:10.2196/27163)
Supplement: Multimedia Appendix 2 [file jmir_v23i8e27163_app2.pdf]

Multimedia Appendix 2

Percentage of patient turnover by days, days of the week (Monday to Sunday) and weekdays vs weekend over a year for five Swiss general hospital types.

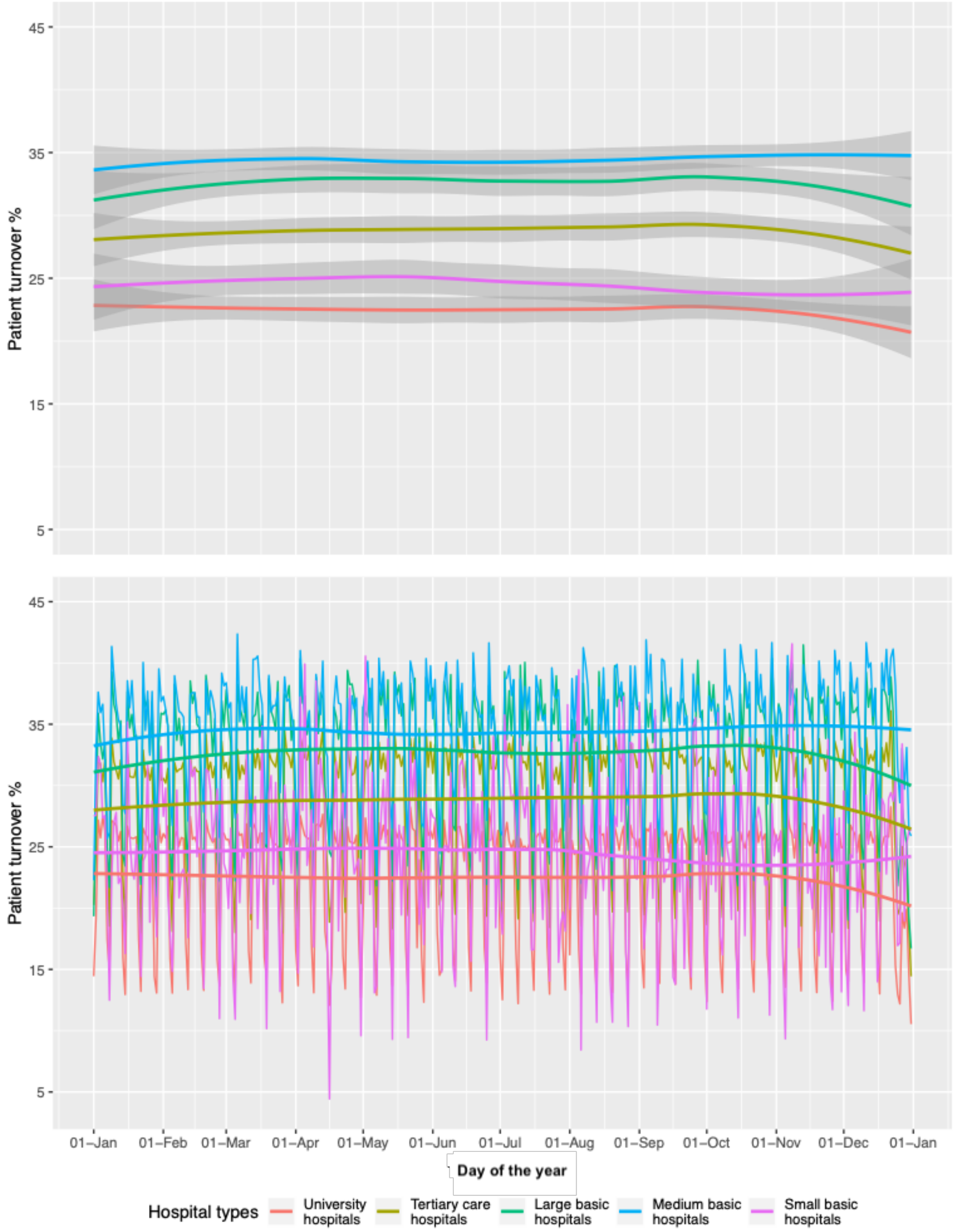

Figure A. Patient turnover of Swiss general hospital types for a year (smooth curve with mean between CI and line chart)

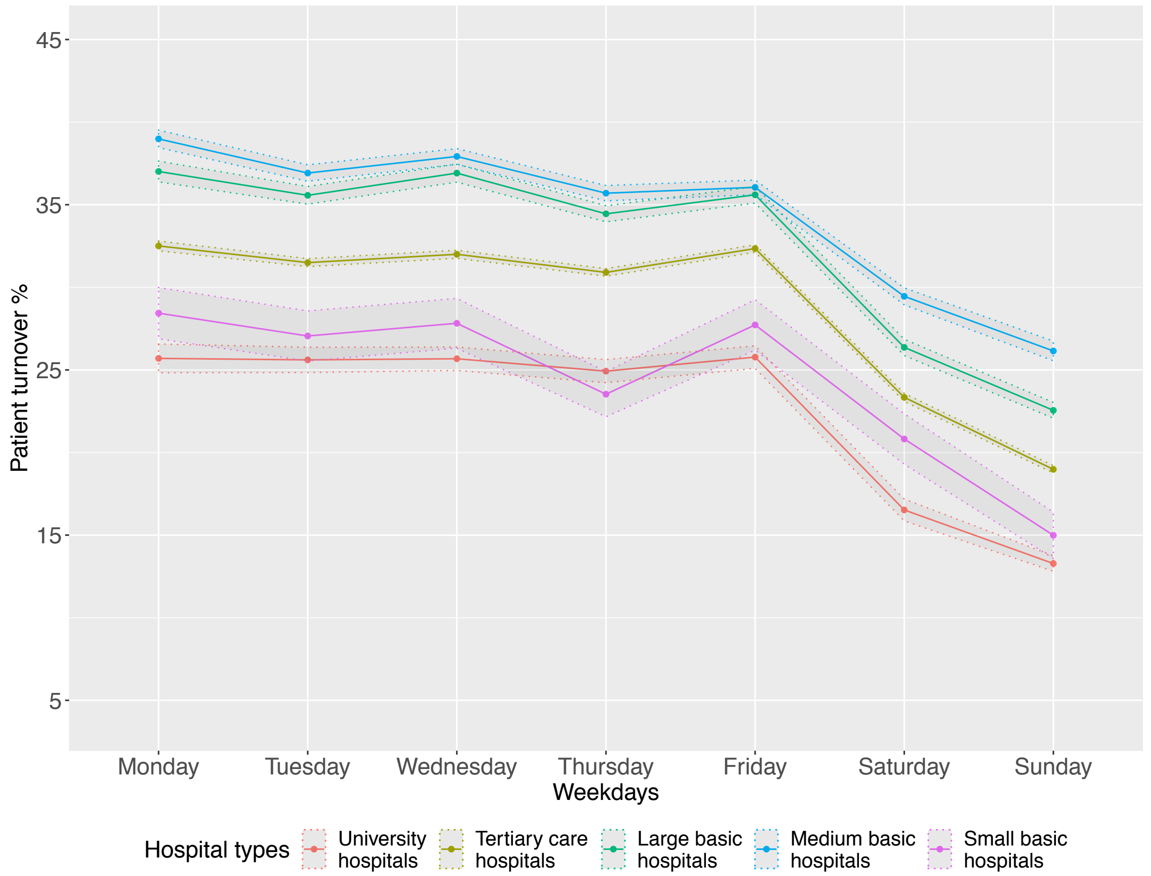

Figure B. Patient turnover of Swiss general hospital types with mean between CI in days of the week

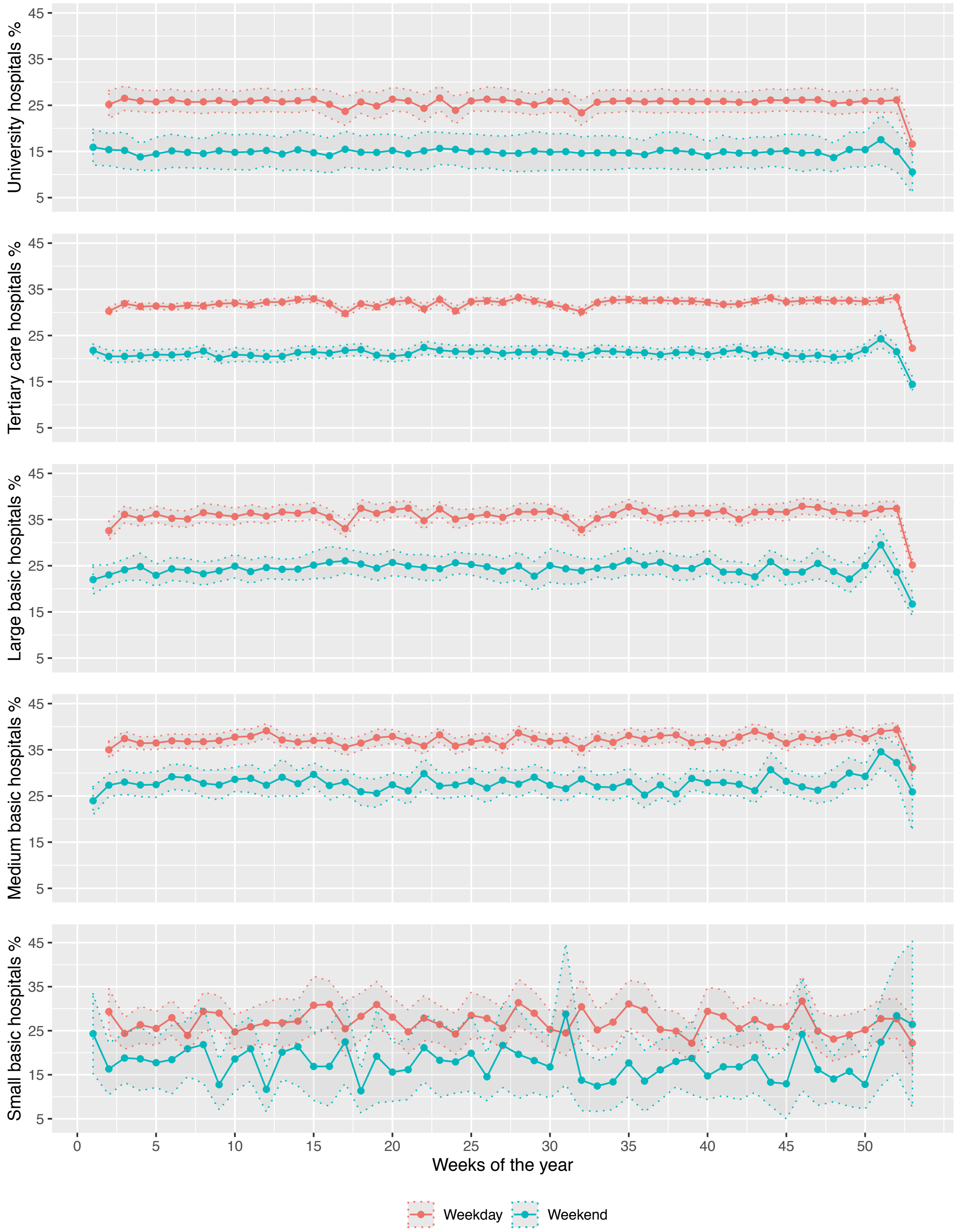

Figure C. Patient turnover of Swiss general hospital types with mean between CI by weekdays and weekend for a year
