# Supplementary material for: Variation of Daily Care Demand in Swiss General Hospitals: Longitudinal Study on Capacity Utilization, Patient Turnover and Clinical Complexity Levels
Source: J Med Internet Res. 2021 Aug 19;23(8):e27163. doi: 10.2196/27163 (PMC8414292; doi:10.2196/27163)
Supplement: Multimedia Appendix 3 [file jmir_v23i8e27163_app3.pdf]

Multimedia Appendix 3

Average patient clinical complexity levels by days, days of the week (Monday to Sunday) and weekdays vs weekend over a year for five Swiss general hospital types.

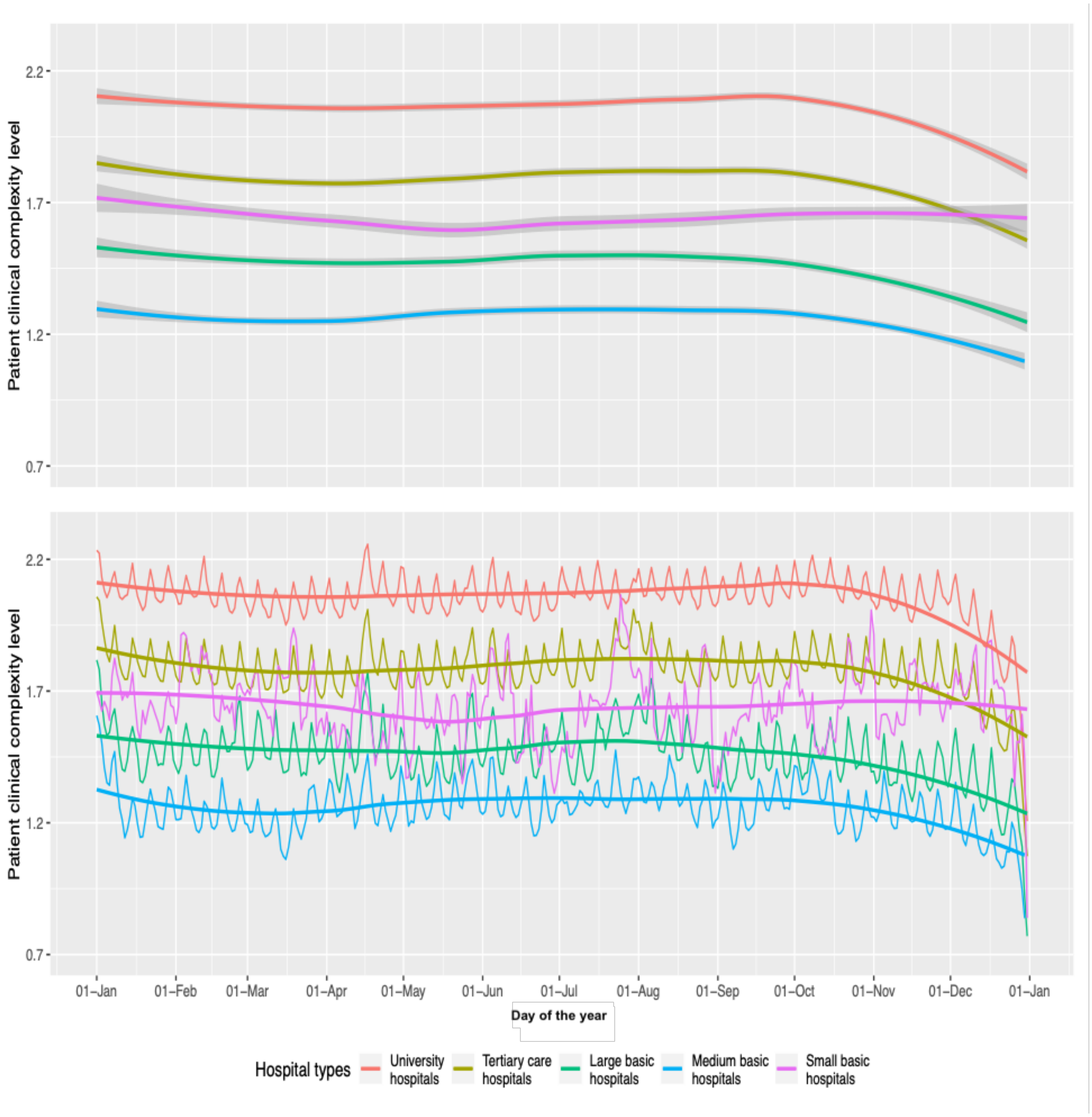

Figure A. Patient clinical complexity level by Swiss general hospital type for one year (smooth curve with mean between CI and line chart)

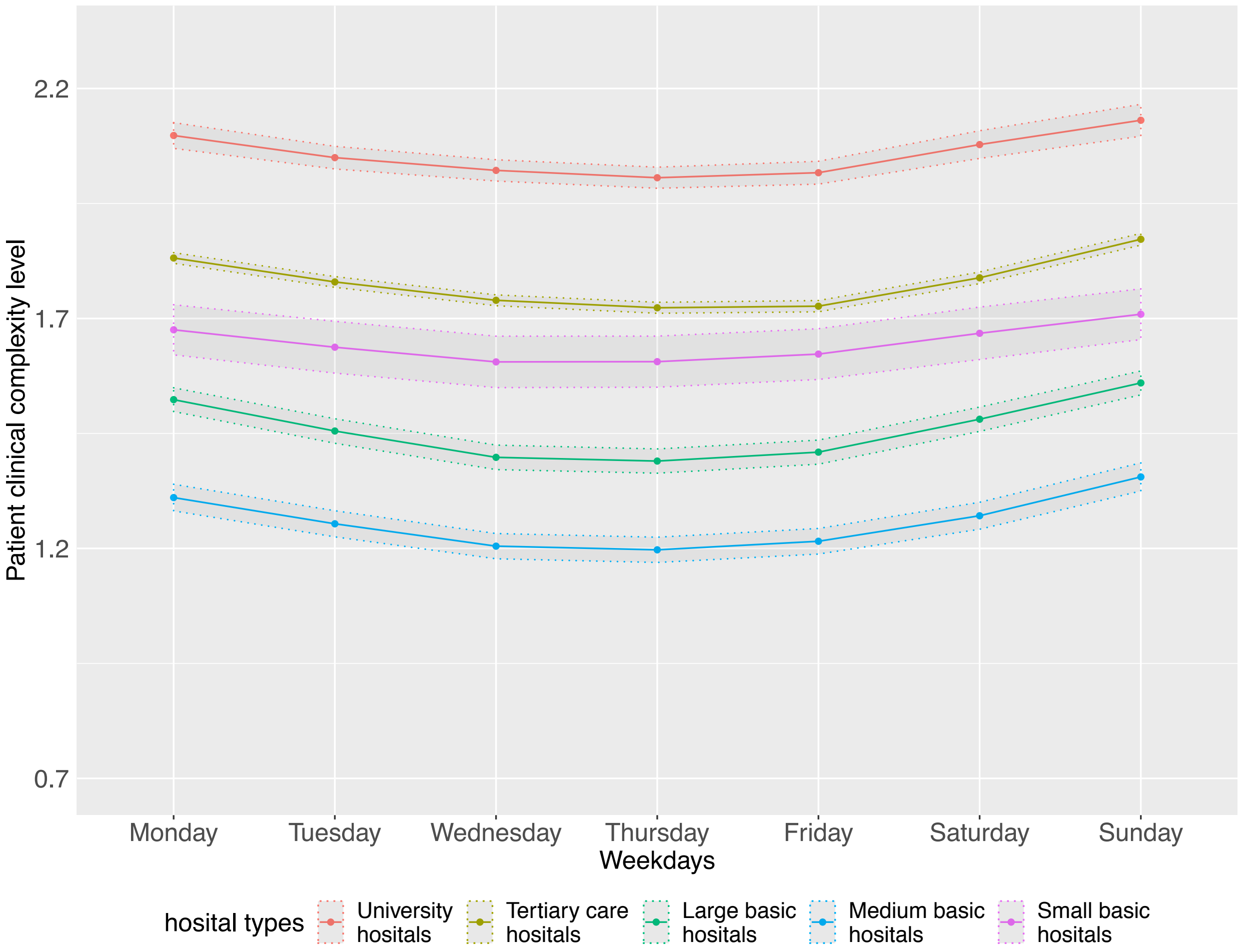

Figure B. Patient clinical complexity level of Swiss general hospital types with mean between CI in days of the week

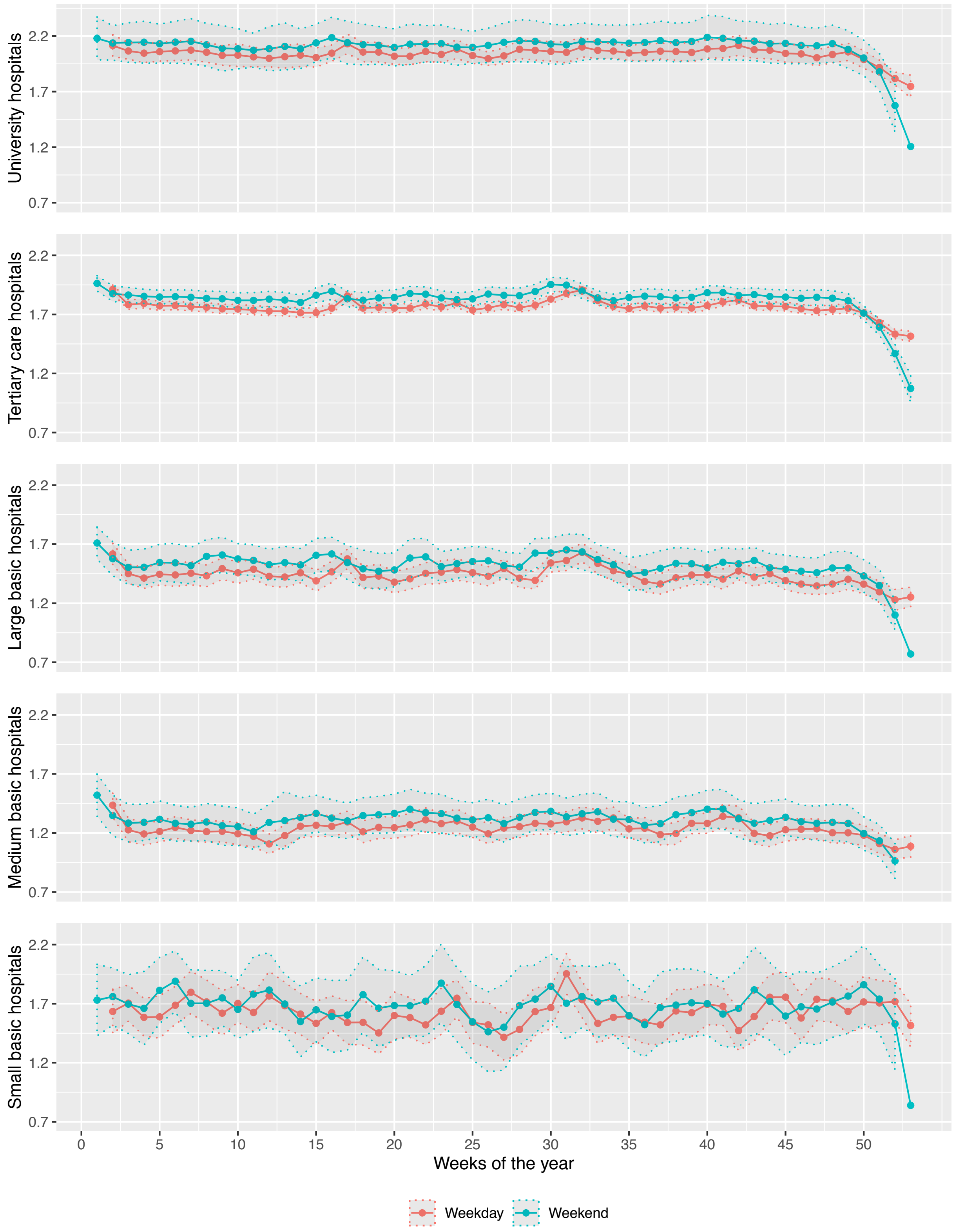

Figure C. Patient clinical complexity level of Swiss general hospital types with mean between CI by weekday and weekend for one year
