# Supplementary material for: Variation of Daily Care Demand in Swiss General Hospitals: Longitudinal Study on Capacity Utilization, Patient Turnover and Clinical Complexity Levels
Source: J Med Internet Res. 2021 Aug 19;23(8):e27163. doi: 10.2196/27163 (PMC8414292; doi:10.2196/27163)
Supplement: Multimedia Appendix 4 [file jmir_v23i8e27163_app4.pdf]

Multimedia Appendix 4

Distribution of patient clinical complexity levels for last 10 weeks of the year with length of stay and average inpatients on weekly basis (showing complex patient stays over Christmas and the end of the year).

Distribution of patient clinical complexity level (last 10 weeks/last 70 days of a study year at a university hospital).

Patient clinical complexity level is calculated only with discharged patients, as ICD-10 diagnosis codes are not available for those who are not discharged.

The mean daily patient clinical complexity level over one study year in a university hospital was 2.38. As the table below shows, patients who were discharged during the last 10 weeks of the year have a lower mean PCCL and shorter length of stay. Thus, the patient clinical complexity level curve drops at the end of the year. Moreover, capacity utilization also drops at the end of the year, due to fewer admissions and more discharges.

This means patients who were admitted but not discharged during the study year might have higher patient clinical complexity level s (severity), warranting stays over the Christmas/New Year period.

Table A. Average daily patient clinical complexity level, LOS and capacity utilization of a university hospital for the last 10 weeks of a study year

Last 10 weeks	Average daily PCCL	Average daily LOS (days)	Average daily inpatients (n)
44	2.27	9.37	918
45	2.22	8.77	929
46	2.21	8.84	915
47	2.19	8.54	944
48	2.23	8.66	942
49	2.27	8.30	893
50	2.17	7.28	883
51	2.03	6.10	838
52	1.94	4.28	644
53	1.78	2.77	277

PCCL: Patient clinical complexity level

Observing Length of stay and Patient clinical complexity level (PCCL) through box plot

Patients with higher PCCLs, e.g., PCCL 4, have longer LOSs (IQR 6-20 days); for those with PCCL 3, LOS IQR = 5-10 days, for PCCLs of 2 and 3, LOS IQR = 2-8 days. I.e., patients not discharged during the end of year period likely have higher PCCLs. Daily PCCLs decline sharply at the end of year.

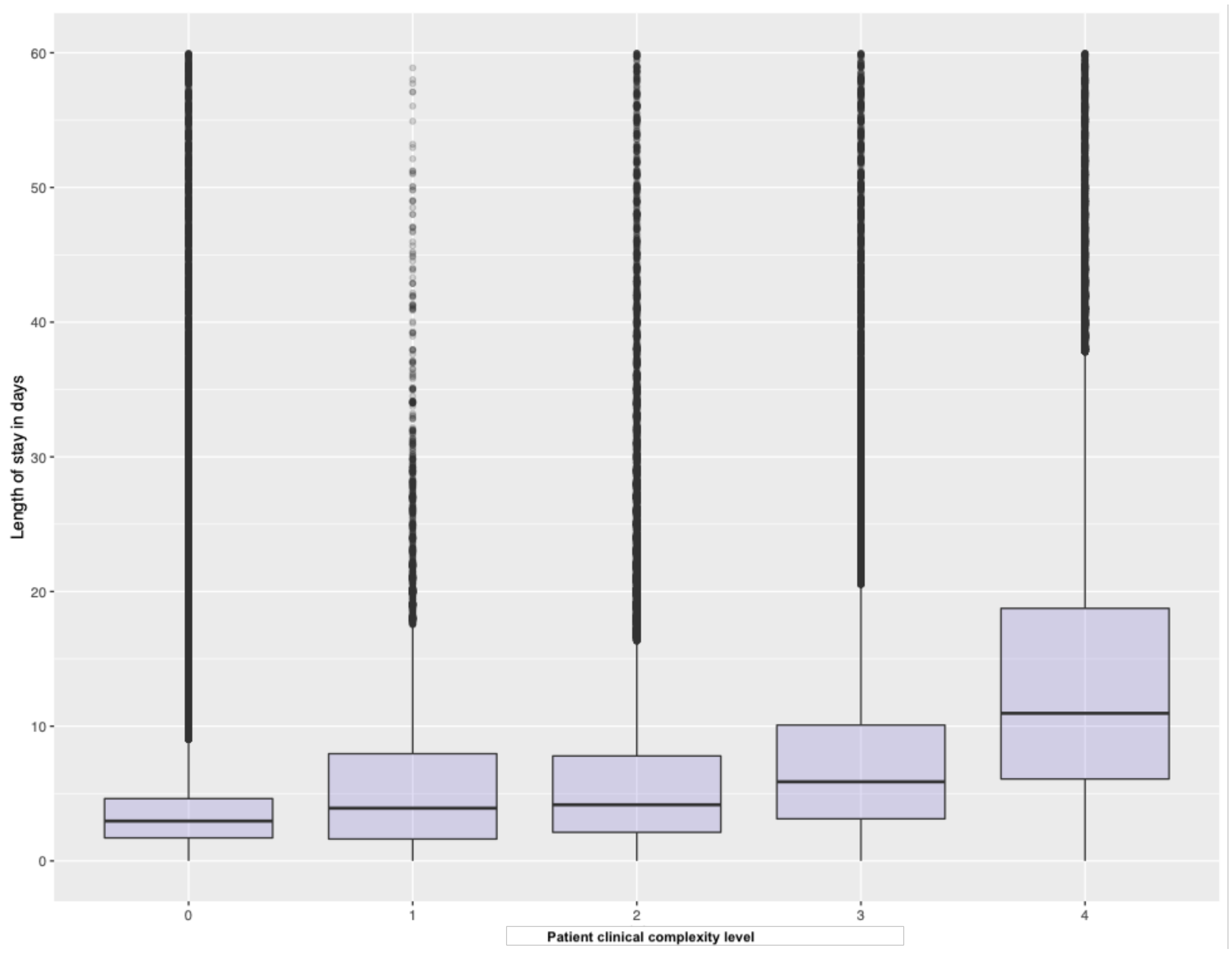

Figure A. Length of stay and patient clinical complexity level (PCCL) values from all discharge data for one study year

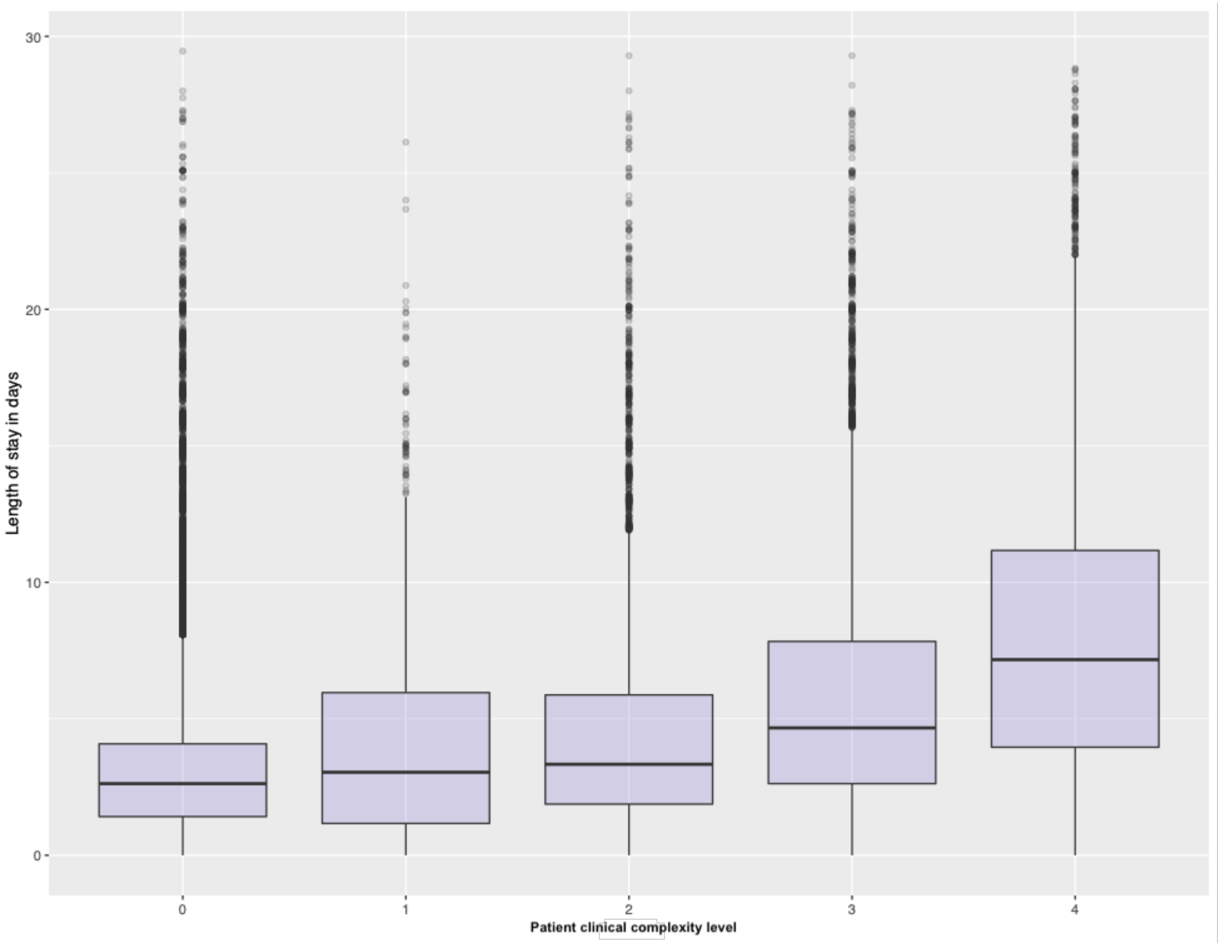

Figure B. Length of stay and Patient clinical complexity level (PCCL) values from all discharge data for November and December
